# The ecological niche of *Dermacentor marginatus* in Germany

**DOI:** 10.1007/s00436-016-4958-9

**Published:** 2016-03-19

**Authors:** Melanie Walter, Katharina Brugger, Franz Rubel

**Affiliations:** Institute for Veterinary Public Health, Department for Farm Animals and Veterinary Public Health, University of Veterinary Medicine Vienna, Veterinärplatz 1, 1210 Vienna, Austria

**Keywords:** Tick map, BIOCLIM, Ecological niche, Habitat model, Q fever

## Abstract

The ixodid tick *Dermacentor marginatus* (Sulzer, 1776) is endemic throughout southern Europe in the range of 33–51 ^°^ N latitude. In Germany, however, *D. marginatus* was exclusively reported in the Rhine valley and adjacent areas. Its northern distribution limit near Giessen is located at the coordinates 8.32 ^°^ E/50.65 ^°^ N. Particularly with regard to the causative agents of rickettsioses, tularemia, and Q fever, the observed locations as well as the potential distribution of the vector *D. marginatus* in Germany are of special interest. Applying a dataset of 118 georeferenced tick locations, the ecological niche for *D. marginatus* was calculated. It is described by six climate parameters based on temperature and relative humidity and another six environmental parameters including land cover classes and altitude. The final ecological niche is determined by the frequency distributions of these 12 parameters at the tick locations. Main parameters are the mean annual temperature (frequency distribution characterized by the minimum, median, and maximum of 6.1, 9.9, and 12.2 ^°^C), the mean annual relative humidity (73.7, 76.7, and 80.9 %), as well as the altitude (87, 240, 1108 m). The climate and environmental niche is used to estimate the habitat suitability of *D. marginatus* in Germany by applying the BIOCLIM model. Finally, the potential spatial distribution of *D. marginatus* was calculated and mapped by determining an optimal threshold value of the suitability index, i.e., the maximum of sensitivity and specificity (Youden index). The model performance is expressed by AUC = 0.91.

## Introduction

**Fig. 1:**
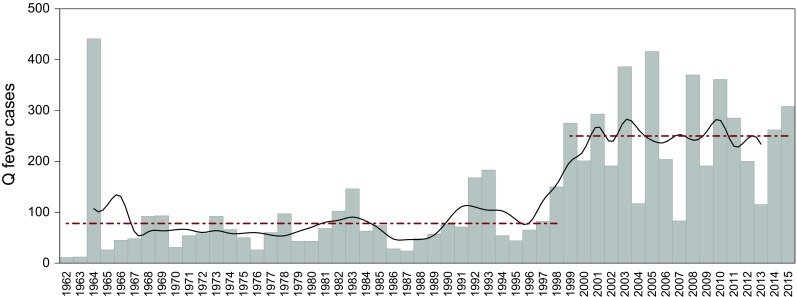
Q fever cases in Germany after Hellenbrand et al. (2001) and Robert Koch-Institut 2015. Human cases (*bars*) with 5-year moving average (*black line*) depict an increase after 1998 by a factor of 3 (*dash-dotted lines*)

In Germany, the ixodid tick *Dermacentor marginatus* (Sulzer, 1976) was exclusively collected in the Rhine-Main valley, frequently from its main host sheep. Associated with the occurrence and spread of Q fever (Fig. 1), *D. marginatus* was recently brought to the focus of public and scientific attention. Because comprehensive studies concerning the occurrence of *Dermacentor* species in Germany are dated several decades back (Liebisch and Rahman 1976), an updated compilation of georeferenced locations as well as an estimation of the potential distribution of *D. marginatus* is presented in this study. For the latter, the ecological niche of *D. marginatus* in Germany was determined, which was also not quantitatively described so far.

*D. marginatus*, the ornate sheep tick, generally inhabits steppes, Alpine steppes, forest steppes, and semi-desert areas. The species is distributed from northern Africa to central Europe up to Iran, Kazakhstan, and the mountain areas of central Asia. Recently published maps based on georeferenced locations were compiled by Estrada-Peña et al. (2013) and Rubel et al. (2016) depicting *D. marginatus* locations in the range of 33–51 ^°^ N latitude. An early map showing the geographical distribution was presented by Hohorst (1943). This map coincides well with current maps except the locations in Great Britain. With great certainty, the latter were ticks of the genus *Dermacentor reticulatus* (Fabricius, 1794), which could not be classified according to modern standards at that time (Schulze 1933). Therefore, historical data should be evaluated with special care in relation to the distinction between *D. marginatus* and *D. reticulatus*.

The life cycle of *D. marginatus* includes three blood meals. Adult females engorged in autumn enter diapause and lay their eggs only in the next spring more or less at the same time as those females engorged in early spring. Engorged larvae and nymphs directly develop to the following life stage without a diapause resulting in a complete generation from egg to adult tick within 1 year. Thus, the development from egg to adult tick might take place in a single growing season. In Germany, this may be reached only along the rivers Rhine and Main, where temperatures are high enough for this thermophilic tick (Kahl and Dautel 2008). Adult ticks have a wide host spectrum and occasionally bite humans. It seems that the main hosts for adults in Germany are sheep (Liebisch and Rahman 1976). Other domesticated hosts are dogs, cattle, goats, and horses. As wild animal hosts deer, hare, hedgehog, wolf, and wild boar have been mentioned. Larvae and nymphs feed primarily on small mammals (Petney et al. 2012) but also birds are mentioned hosts (Farkas et al. 2013). Adult ticks are active during spring and early summer as well as in autumn (Nosek 1972). Hornok and Farkas (2009) reported the highest numbers of *D. marginatus* in February and March, which coincides with the maximum of *D. marginatus*-related disease cases (Oteo and Portillo 2012).

*D. marginatus* is a potential or proven vector of various zoonotic diseases. In Germany, pathogens detected in *D. marginatus* comprise rickettsiae of the spotted fever group, i.e., *Rickettsia slovaca* and *R. raoulti* (Rehácek et al. 1977; Pluta et al. 2010) as well as *Coxiella burnetii*, the causative agent of Q fever (Liebisch et al. 1978). The natural cycle of the Q fever agent is between *D. marginatus* and rodents. But also larger animals, mainly sheep, were infected and are capable of spreading these bacterial particles to the environment resulting in infections of humans (Sting et al. 2004). Feeding *Dermacentor* discharge large amounts of feces. The dried feces contain *C. burnetii* spores and are taken by the wind and spread. This seems to be the main infectious route of *C. burnetii* with *Dermacentor* involved. In Europe, even more zoonotic pathogens were proven in this tick species. Bonnet et al. (2013) reported *D. marginatus* ticks infected with *Anaplasma phagocytophilum*, *A. marginale*, *Bartonella* spp., *Theileria* spp., and *Francisella philomiragia* in France. The causative agent of equine piroplasmosis, *T. equi*, was detected in Italy (Iori et al. 2010). *D. marginatus* is also known to be infected with viral pathogens like the tick-borne encephalitis virus (Hubálek and Rudolf 2012). In Europe, *D. marginatus* is in focus because of its vector function for *R. slovaca* and *R. raoulti* causing tick-borne-lymphadenopathy, TIBOLA (Parola et al. 2009; Oteo and Portillo 2012). The first human TIBOLA cases in Germany were reported from Freiburg and southern Rhineland-Palatinate (Pluta et al. 2009; Rieg et al. 2011), where *D. marginatus* as well as *R. slovaca* and *R. raoulti* have been observed (Rehácek et al. 1977; Pluta et al. 2009). As a consequence of the potential expansion of *D. marginatus* in the course of global warming (Hartelt et al. 2008), TIBOLA may also expand northwards.

Here, parameters derived from climate, land cover, and altitude were used to determine the ecological niche of *D. marginatus*. To this end, empirical frequency distributions of these parameters at all tick occurrence locations were calculated. Statistical quantities of these frequency distributions were not only used to quantify the environmental envelope of the tick habitats but also to model the potential distribution of *D. marginatus* in Germany. The first model to estimate species distribution was the BIOCLIM algorithm (Busby 1986; Guisan and Zimmermann 2000), which is applied here. All other models characterizing the climatological niche of species by abiotic factors are based on this method. According to Hijmans and Elith (2015), the large number of algorithms can be divided into three groups: profile methods (e.g., BIOCLIM), regression models and machine learning algorithms. The maximum entropy algorithm MaxEnt (Phillips and Dudík M 2008), which can be assigned to the machine learning models, is the latest developmental stage of these models and presently most often applied to estimate the potential distribution of ticks, for example by Estrada-Peña et al. (2013) and Feria-Arroyo et al. (2014). BIOCLIM, however, was mainly used to estimate the distribution of plant species. One of few BIOCLIM implementations concerning ticks was presented by Jackson et al. (2007) to describe the spread of *Ixodes cornuatus* and *I. holocyclus* in southeast Australia. Here, preference is given to BIOCLIM because it guarantees the full understanding and control of the method to the user and, as a by-product, explicitly defines the ecological niche based on environmental parameters. Finally, it is demonstrated that BIOCLIM results in a satisfactory and reliable *D. marginatus* distribution at the regional scale.

## Data and methods

### Tick occurrence data

Georeferenced occurrences of the ixodid tick *D. marginatus* were taken from the dataset compiled by Rubel et al. (2014). This dataset comprises 77 locations digitized from historical documentations (Liebisch and Rahman 1976) as well as recent observations from Menn (2006). For this study, 41 additional locations (Table 1) were collected resulting in a total of 118 georeferenced *D. marginatus* locations. However, locations described by geographical coordinates determined by GPS in the field were only provided by studies carried out after the turn of the millennium (Menn 2006; Dries 2012; Gilgenast 2013; Kahl 2015). Thus, accuracy measures were given for all data referenced in Table 1. It is distinguished between high (h), medium (m), low (l), and unknown (u) accuracy. A high accuracy (±0.1 km) was allocated to coordinates given in degrees, minutes, and seconds or in decimal degrees with at least 4–5 relevant decimal places. A medium accuracy (± 1 km) was assumed for coordinates given in degrees and minutes or in decimal degrees with at least 2–3 relevant decimal places. A medium accuracy was also assumed for ticks collected from animals or humans and for coordinates digitized from local maps (<1000 km ^2^). Coordinates digitized from regional maps (>1000 km ^2^) were classified as low-accuracy data (±10 km).

**Table 1:** Georeferenced *Dermacentor marginatus* locations from Germany and the border areas of France, Switzerland and Austria comprising geographical longitude, latitude, accuracy and source. Note that further locations used in this paper have been documented by Rubel et al. (2014)

No.	Longitude	Latitude	Accuracy	Source
1	8.4800	49.1336	h	Kahl (2015)
2	8.3750	49.1353	h	Kahl (2015)
3	7.9608	48.4442	h	Kahl (2015)
4	9.1144	48.5850	h	Kahl (2015)
5	9.1246	49.9785	m	Pluta et al. (2010)
6	7.6796	47.6187	m	Pluta et al. (2010)
7	8.1324	49.0529	h	Gilgenast (2013)
8	8.0308	49.0351	h	Gilgenast (2013)
9	7.8933	48.4627	h	Dries (2012)
10	7.5000	48.5000	u	Estrada-Peña (2013)
11	7.7500	48.5833	u	Estrada-Peña (2013)
12	8.3799	49.2164	u	Foley et al. (2014)
13	11.3720	47.2700	m	Thaler (2003)
14	10.8590	47.2195	m	Thaler (2003)
15	10.3818	46.8363	m	Thaler (2003)
16	11.2722	47.2666	m	Thaler (2003)
17	7.5539	47.6285	l	Immler et al. (1970)
18	7.2863	47.8130	l	Immler et al. (1970)
19	7.4123	47.9852	l	Immler et al. (1970)
20	7.5680	47.6709	l	Sting et al. (2004)
21	7.6498	47.6710	l	Sting et al. (2004)
22	8.4105	47.7982	l	Sting et al. (2004)
23	7.8218	48.0333	l	Sting et al. (2004)
24	7.7189	48.1334	l	Sting et al. (2004)
25	7.7280	48.1946	l	Sting et al. (2004)
26	8.0096	48.4787	l	Sting et al. (2004)
27	7.8573	48.5147	l	Sting et al. (2004)
28	7.8632	48.5733	l	Sting et al. (2004)
29	8.0351	48.6441	l	Sting et al. (2004)
30	8.1807	48.6636	l	Sting et al. (2004)
31	8.2548	48.6568	l	Sting et al. (2004)
32	8.9929	48.4141	l	Sting et al. (2004)
33	9.5154	48.7082	l	Sting et al. (2004)
34	8.6191	48.8675	l	Sting et al. (2004)
35	8.6278	48.9718	l	Sting et al. (2004)
36	8.8523	48.9577	l	Sting et al. (2004)
37	8.5510	49.1036	l	Sting et al. (2004)
38	8.4922	49.1160	l	Sting et al. (2004)
39	9.1020	49.1084	l	Sting et al. (2004)
40	9.1911	49.0858	l	Sting et al. (2004)
41	9.2612	49.0225	l	Sting et al. (2004)

Note that Table 1 contains not only tick locations from Germany but also three observations from the Inn river valley in Austria (Thaler 2003) as well as five observations from the French and Swiss part of the Rhine river valley (Immler et al. 1970; Estrada-Peña et al. 2013).

### Environmental data

In order to characterize the abiotic habitat of the tick, predictive environmental data comprising climate, land cover, and altitude parameters were used. These gridded data cover the entire region of Germany defined by the rectangular model domain ranging from 5.5 ^°^ E to 15.5 ^°^ E longitude and 47.0 ^°^ N to 55.5 ^°^ N latitude. A grid spacing of 0.1 ^°^, corresponding to a cell size of about 80 km ^2^, was selected in accordance to the spatial resolution of the available environmental data. Thus, the model domain is covered by a 85 · 100 grid.

**Fig. 2:**
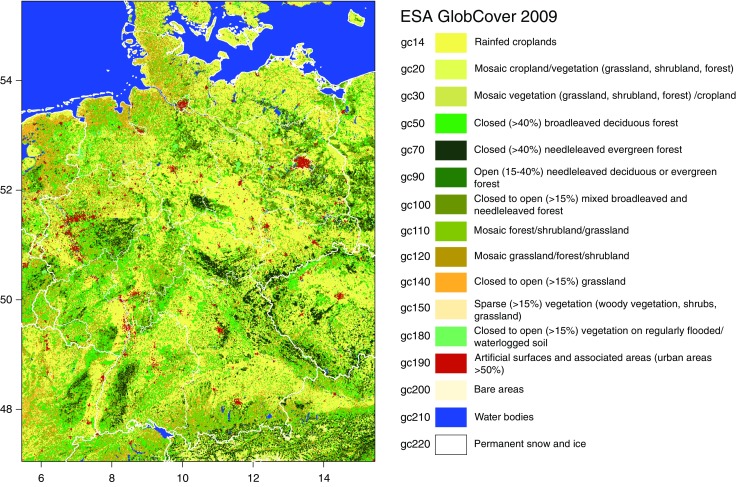
Model domain with land cover provided by European Space Agency (2010)

Climate parameters were derived from temperature and relative humidity data provided by the German Weather Service. Here, the so-called HYRAS dataset from the KLIWAS (*Impacts of climate change on waterways and navigation - searching for options of adaptation*) project is used (Rauthe et al. 2013; Frick et al. 2014). Land cover parameters were taken from the ESA *GlobCover 2009* dataset (European Space Agency 2010). It characterizes, specified by satellite images, the land cover based on 22 classes. A total of 16 land cover classes were found in the model domain depicted in Fig. 2. The original spatial resolution of these categorical data is 300 m. They were aggregated to the grid spacing of 0.1 ^°^ described above by determining the fraction of the land cover classes within each grid cell. By applying this procedure, the categorical land cover classes were converted to continuous data, i.e., fractions of land cover classes within a grid cell. The latter were used as predictive variables in the BIOCLIM model.

### BIOCLIM algorithm

The bioclimatic analysis and prediction system BIOCLIM (Busby 1986, 1991) is used to investigate the ecological niche of *D. marginatus* in Germany. BIOCLIM may also be characterized as the first and most widely known habitat model or as the classical climate-envelope model. Originally, BIOCLIM creates a climatic profile of the abiotic habitat of a species, the so-called climatic envelope. It is defined by the minimum and maximum of each environmental parameter within the subset of all grid cells where the species was observed, which then form a climatic minimum rectangular envelope in a more-dimensional space (Di Febbraro and Mori 2015). Here, not only climate but also land cover and altitude parameters were used, and the resulting environmental envelope is assumed to statistically describe the ecological niche. Note that environmental parameters determining the ecological niche should be selected according to the resolved scale. The smaller the scale, the more important are biotic interactions. Relevant parameters on the regional scale considered here are climate, topography, and land use (Pearson and Dawson 2003; Boehnke et al. 2015).

Knowing the ecological niche, i.e., the frequency distributions of the environmental parameters determined from all *D. marginatus* locations, the habitat suitability may be calculated (Hijmans and Graham 2006). For a grid cell and a single parameter, the maximal suitability index of si = 1 is calculated for values equal to the median of the frequency distribution. If the parameter value of a grid cell is outside the range defined by the minimum and maximum, the suitability index equals si = 0. Generally, the suitability index is derived from the percentile rank of a parameter at a specific grid cell. The final suitability index of a grid cell is defined as the lowest suitability index of all environmental parameters (Liebig’s law of the minimum). Mapping the suitability indices depicts the potential distribution of a species.

## Results

### Ecological niche

**Fig. 3:**
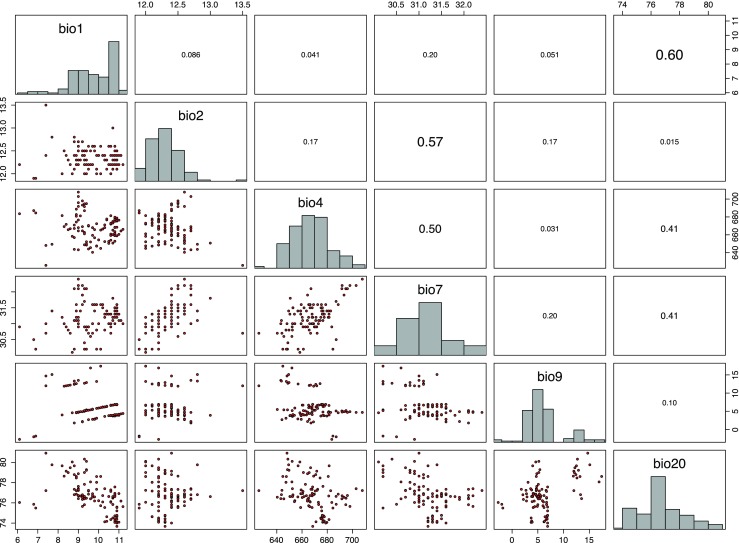
Selected five bioclimatic parameters of temperature (bio1, bio2, bio4, bio7, bio9) and one bioclimatic variable of relative humidity (bio20). *Diagonal*: frequency distributions. *Lower left*: scatter plots. *Upper right*: Spearman correlation coefficients depicting low collinearity. Parameters correlated above *r*_S_=0.66 were removed

**Fig. 4:**
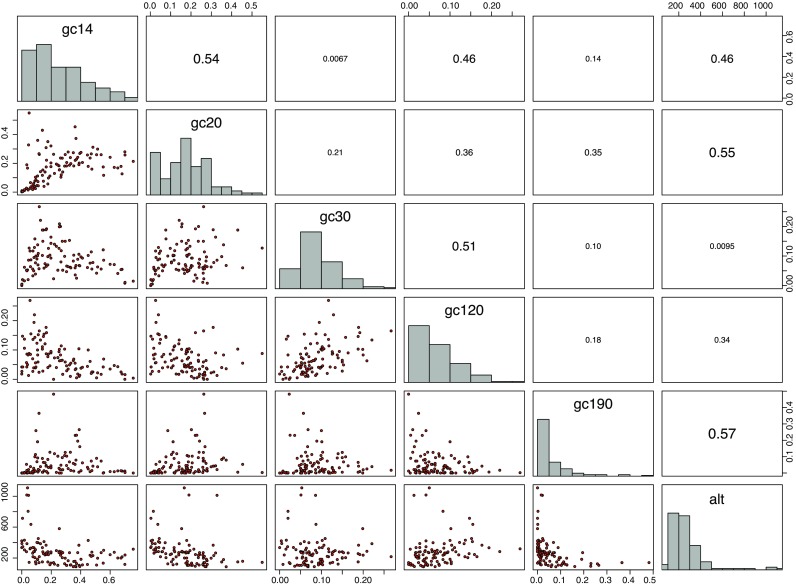
Selected five landcover classes (gc14, gc20, gc30, gc120, gc190) and altitude (alt). *Diagonal*: frequency distributions. *Lower left*: scatter plots. *Upper right*: Spearman correlation coefficients depicting low collinearity. Parameters correlated above *r*_S_=0.66 were removed

The most common climate classification worldwide (Rubel and Kottek 2011) was developed in the first half of the twentieth century by the German climatologists Wladimir Köppen and Rudolf Geiger, recently updated and projected to climate change scenarios by Kottek et al. (2006) and Rubel and Kottek (2010). According to that Köppen-Geiger climate classification, the geographical distribution of *D. marginatus* (Rubel et al. 2016) is restricted to warm temperate climates which may be fully humid with warm summers(Cfb) or summer dry with warm (Csb) and hot (Csa) summers. The northern distribution limit of *D. marginatus* is located in Germany near Giessen at the coordinates 8.32 ^°^ E/50.65 ^°^ N. Because almost the entire region of Germany is covered by the Cfb climate, which is characterized by less hot and dry summers compared to the climate of the main distribution area in the Mediterranean countries, it is necessary to specify the climate and environmental conditions in more detail to capture the observed distribution of *D. marginatus*. Therefore, the 19 well-known bioclimatic parameters (Busby 1986) were calculated from temperature and relative humidity data provided by the German weather service (Frick et al. 2014; Rauthe et al. 2013). Together with the 16 ESA land cover classes (Fig. 2) and the altitude, a total of 36 parameters were investigated to determine the ecological niche of the ticks. Parameters highly correlated with each other were eliminated to reduce the number of parameters as well as to avoid collinearity. Therefore, a threshold Spearman correlation coefficient of *r*_S_ = 0.66 was applied to specify the minimum number of climate parameters necessary for high accuracy habitat modeling (see next section). This threshold was empirically specified from sensitivity analysis (Walter 2015) and is in good agreement with thresholds applied by other authors (Melaun et al. 2015). The correlation analysis results in six bioclimatic parameters (Fig. 3) and six environmental parameters (Fig. 4) selected to define the ecological niche of *D. marginatus* in Germany. In Table 2, these 12 parameters are listed together with the median, the minimum, and the maximum derived from their frequency distributions. The bioclimatic parameter bio1, for example, represents the mean annual temperature characterized by the minimum, median, and maximum of 6.1, 9.9, and 12.2 ^°^C, bio2 represents the mean diurnal temperature range (11.6, 12.3, and 13.5 ^°^C), etc. Further important parameters comprise the mean annual relative humidity (73.7, 76.7, and 80.9 %), the fraction of rainfed croplands in a grid cell (0, 0.22, 0.75), the mosaic cropland/vegetation (0, 0.18, 0.55) as well as the altitude (87, 240, 1108 m).

**Table 2:** Parameters defining the climate and environmental profile by median, minimum and maximum values. Temperatures bio1, bio2, bio4, bio7, and bio9 are given in ^°^C, relative humidity bio20 is given in %, fractions of land classifications in each grid cell gc14, gc20, gc30, gc120, and gc200 are dimensionless and the altitude alt is given in meters above sea level

Abbr.	Climate and environmental parameters	Min	Median	Max
bio1	Mean annual temperature	6.1	9.9	11.2
bio2	Mean diurnal temperature range	11.9	12.3	13.5
bio4	Temperature seasonality	6.3	6.7	7.1
bio7	Temperature annual range	30.1	31.2	32.4
bio9	Mean temperature of the driest quarter	-2.7	5.1	17.4
bio20	Mean annual relative humidity	73.7	76.7	80.9
gc14	Fraction of rainfed croplands	0	0.222	0.757
gc20	Mosaic cropland/vegetation (grassland, shrub land, forest)	0.002	0.179	0.550
gc30	Mosaic vegetation (grassland, shrub land, forest)/cropland	0	0.077	0.267
gc120	Mosaic forest/shrub land/grassland	0	0.057	0.269
gc190	Artificial surfaces and associated areas (urban areas >50 %)	0	0.025	0.482
alt	Altitude	87	240	1108

### Potential species distribution

**Fig. 5:**
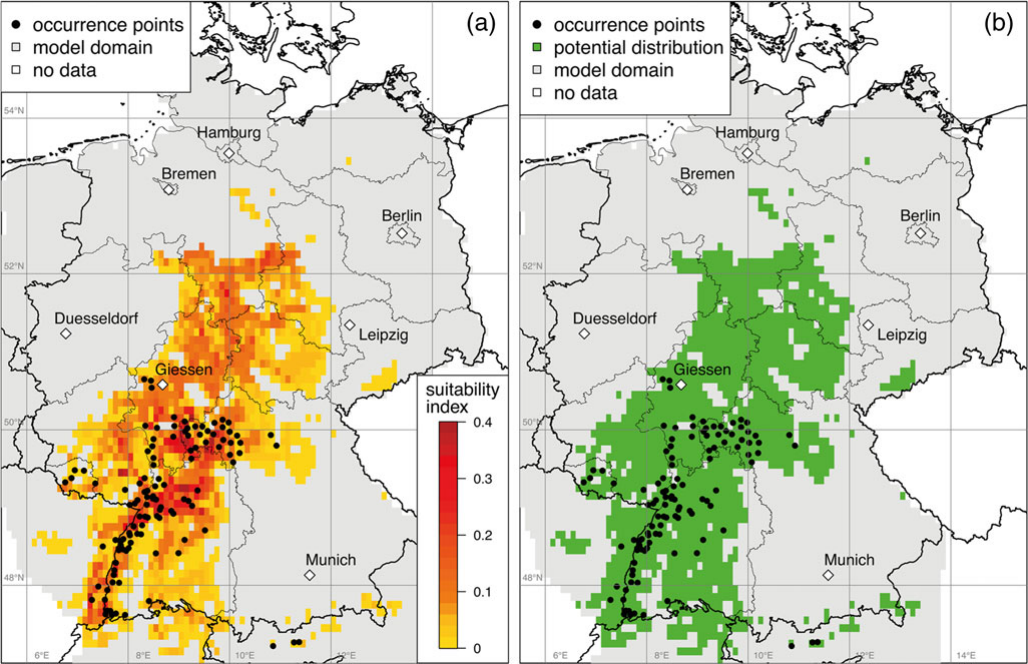
Distribution of *Dermacentor marginatus* in Germany. Suitability index (**a**) and potential distribution (**b**) with overlayed occurrence points

For the estimation of the potential species distribution of *D. marginatus* in Germany, the BIOCLIM model was applied together with the 12 parameters (predictive variables) described above. Figure 5a depicts the resulting map of the habitat suitability index (si) overlayed by the observed 118 tick locations (occurrence points). To categorize those regions where the occurrence of *D. marginatus* is most likely, a threshold for the suitability index of si = 0.01 was determined. From several methods proposed to estimate an optimum threshold, the maximum of sensitivity plus specificity (Youden index) was selected (Liu et al. 2005; Hijmans and Elith 2015). Figure 5b shows the resulting region identified as potential distribution area for *D. marginatus*. It covers 1508 out of 5770 grid cells (about 27 %) of the model domain in the south-western and central part of Germany as well as adjacent regions in France, Switzerland, and the Austrian Inn valley. The modeled *D. marginatus* habitats in Germany not only cover largely the region of known occurrence along the Rhine and Main rivers (Baden-Württemberg, Rhineland-Palatinate, Saarland, Hesse, Bavaria) but also extend beyond the east and the north (especially the northern parts of Hesse, North Rhine-Westphalia, Thuringia, Lower Saxony, and Saxony-Anhalt). It is notable that the northernmost populations of *D. marginatus* were observed at about 51 ^°^ N (near Giessen), while a northern distribution limit of 53 ^°^ N and beyond was modeled (Fig. 5).

The model performance is expressed by the commonly used measure *Area Under the Receiver Operated Curve* (AUC; Liu et al. 2011; Hijmans and Elith 2015). The calculated value of AUC = 0.91 is indicative of a good model formation and similar to those of the MaxEnt model for the arbovirus vector *Ochlerotatus japonicus japonicus* in Germany (Melaun et al. 2015). The latter showed a distribution similar to that of the ixodid tick *D. marginatus*. This is the case at least for the Rhine-Main valley with its warm and dry summers, which is known for the occurrence of various Mediterranean animal and plant species.

## Discussion and outlook

The Rhine-Main valley is located at the northern limit of the current range of the thermophilic tick *D. marginatus*, whose potential distribution was estimated by a habitat model. Thereby, the BIOCLIM model has been preferred over more sophisticated models such as Maxent (Phillips and Dudík M 2008; Schapire 2014) because it is the first and most widely used habitat model; its implementation provides the full access option concerning the model algorithm and not least performs well on the regional scale. In general, the importance of the selected algorithm, i.e., the selected habitat model, decreases with increasing quality of the input data. The latter comprise both the reliability and number of tick occurrence sites as well as the quality of the predictive variables. Comparisons of model algorithms and datasets support this thesis (De Clercq et al. 2015).

Conversely, even the most developed habitat models are not able to predict complete and detailed species distributions if they are based on inadequate data. It is essential that the area of distribution of the target tick has been thoroughly and homogeneously surveyed and that all the possible combinations of climate under which the tick has stable populations are considered (Estrada-Peña et al. 2014). Otherwise habitat models have serious problems to depict the species distribution in unsampled regions or to project future species distributions based on climate change scenarios. The *D. marginatus* distribution maps from Williams et al. (2015), estimated with the MaxEnt model, are a good example of this. The model failed to depict the known habitat suitability in Germany (Rubel et al. 2014), Portugal (Santos-Silva et al. 2011), and Romania (Mihalca et al. 2012). As a consequence, also the *D. marginatus* maps projected to climate change scenarios are unrealistic.

In this study, special focus was put on the collection and application of best available data. The 77 *D. marginatus* locations recently collected by Rubel et al. (2014) were complemented by 41 additional locations and the climate parameters (Table 2) were calculated from the national climate database. These regional scale data of the German weather service (Frick et al. 2014; Rauthe et al. 2013) are based on more and more recent ground measurements than the most widely applied climate data provided by Hijmans et al. (2005), which are unrivaled on the global and continental scale. Further, except for the land cover classification, satellite-derived data were not considered. Remotely sensed climate parameters were generally indirectly derived from radiation measurements implying lower accuracy and artefacts (Rubel and Rudolf 2001) or systematic errors (Alonso-Carné et al. 2013), so ground-based measurements were preferred.

A weak point in the available tick observations is that they were mainly collected from sheep, although it is well known that *D. marginatus* may be found on various other hosts, especially on wildlife. Also dogs should be taken into consideration to be hosts for *D. marginatus* as may be deduced from the study by Wächter et al. (2015), who detected antibodies against *R. raoultii* and *R. slovaca* in dogs from the south and southwest of Germany. The results presented should therefore be taken as a basis to perform a new study concerning the species distribution and the vector potential of *D. marginatus*. The latest germanwide study took place more than 40 years ago (Liebisch and Rahman 1976), and the modeled distribution (Fig. 5) provides evidence that *D. marginatus* extended its range northwards and westwards. In these regions, exclusively, *D. reticulatus* have been described, but a misclassification cannot be excluded.
